# Mobile Health Divide Between Clinicians and Patients in Cancer Care: Results From a Cross-Sectional International Survey

**DOI:** 10.2196/13584

**Published:** 2019-09-06

**Authors:** Rosanna Tarricone, Maria Cucciniello, Patrizio Armeni, Francesco Petracca, Kevin C Desouza, Leslie Kelly Hall, Dorothy Keefe

**Affiliations:** 1 Department of Social and Political Science Bocconi University Milan Italy; 2 Centre for Research on Health and Social Care Management SDA Bocconi School of Management Bocconi University Milan Italy; 3 School of Management QUT Business School Queensland University of Technology Brisbane Australia; 4 Healthwise Boise, ID United States; 5 Engaging Patient Strategy Boise, ID United States; 6 School of Medicine Faculty of Health Sciences University of Adelaide Adelaide Australia

**Keywords:** mHealth, cancer, mobile phone, survey, mobile app, digital health, mhealth

## Abstract

**Background:**

Mobile technologies are increasingly being used to manage chronic diseases, including cancer, with the promise of improving the efficiency and effectiveness of care. Among the myriad of mobile technologies in health care, we have seen an explosion of mobile apps. The rapid increase in digital health apps is not paralleled by a similar trend in usage statistics by clinicians and patients. Little is known about how much and in what ways mobile health (mHealth) apps are used by clinicians and patients for cancer care, what variables affect their use of mHealth, and what patients’ and clinicians’ expectations of mHealth apps are.

**Objective:**

This study aimed to describe the patient and clinician population that uses mHealth in cancer care and to provide recommendations to app developers and regulators to generally increase the use and efficacy of mHealth apps.

**Methods:**

Through a cross-sectional Web-based survey, we explored the current utilization rates of mHealth in cancer care and factors that explain the differences in utilization by patients and clinicians across the United States and 5 different countries in Europe. In addition, we conducted an international workshop with more than 100 stakeholders and a roundtable with key representatives of international organizations of clinicians and patients to solicit feedback on the survey results and develop insights into mHealth app development practices.

**Results:**

A total of 1033 patients and 1116 clinicians participated in the survey. The proportion of cancer patients using mHealth (294/1033, 28.46%) was far lower than that of clinicians (859/1116, 76.97%). Accounting for age and salary level, the marginal probabilities of use at means are still significantly different between the 2 groups and were 69.8% for clinicians and 38.7% for patients using the propensity score–based regression adjustment with weighting technique. Moreover, our analysis identified a gap between basic and advanced users, with a prevalent use for activities related to the automation of processes and the interaction with other individuals and a limited adoption for side-effect management and compliance monitoring in both groups.

**Conclusions:**

mHealth apps can provide access to clinical and economic data that are low cost, easy to access, and personalized. The benefits can go as far as increasing patients’ chances of overall survival. However, despite its potential, evidence on the actual use of mobile technologies in cancer care is not promising. If the promise of mHealth is to be fulfilled, clinician and patient usage rates will need to converge. Ideally, cancer apps should be designed in ways that strengthen the patient-physician relationship, ease physicians’ workload, be tested for validity and effectiveness, and fit the criteria for reimbursement.

## Introduction

### Background

Many would agree that mobile health (mHealth; the use of portable devices for medical purposes) holds considerable promise for improving health care and the quality of life for people with cancer [1]. With internet access growing worldwide and well over 70% of people in Europe and the United States owning smartphones, the potential market for mHealth is very large and is projected to continue to grow [2].

In 2017, there were 325,000 mHealth (health and fitness and medical) apps with over 3.7 billion downloads [3], reflecting over 30% growth compared with 2016. Although the number of apps for wellness management decreased by 18% from 2015 to 2017, the number for managing health conditions increased by 48% in the same period [3].

Although the potential benefits of mHealth seem particularly compelling for managing chronic conditions, where the overall efficacy largely depends on patient compliance that frequently occurs outside of the formal health care system, prolonged, regular, and intensive use still represents a major challenge [4]. Apps specifically developed for chronic disease management have had some initial success but have so far failed to live up to their expectations [5-7]. Among specific diseases, the greatest proportion of apps on the market is for mental health and behavioral disorders (28%), followed by diabetes (16%) and cardiovascular disorders (11%) [3]. Although nearly 1 in 6 deaths are due to cancer, which is indeed among the leading causes of mortality (with an estimated 9.6 million deaths in 2018 and approximately 14 million new cases worldwide every year, projected to increase up to 22 million within the next two decades) [8], few of the mHealth apps focus on cancer care (5%). Not only are mHealth cancer apps relatively few, but the action put forward thus far has not been steered in the right direction; the available cancer apps mostly focus on awareness raising and information provision [9] and appear to be used for limited purposes in the actual health care process, with a prevailing focus on self-management activities and the automation of structured and unstructured processes [10]. Although the improved cancer survival rates and outcomes have led to considering most cancers as chronic, their treatment is still accompanied by distressing symptoms and serious toxicities that affect functioning and quality of life [11]. To address these issues, mHealth has the potential to track the patient experience and collect patient-reported outcomes to personalize care, draw insights, and shorten the cycle from research to clinical implementation [12].

When patients are able to record their experiences in real time and combine them with passive data collection from sensors and mobile devices, this information can inform better care for each patient and contribute to the growing body of health data that can be used to draw insights for all patients.

Preliminary research has addressed the interest of cancer patients in the use of mobile technologies to manage their disease [13,14], whereas the influence of demographic factors on predicting the use of Web-based health information resources and its patterns has been mostly assessed with respect to electronic health technologies [15-17]. Furthermore, unlike other medical devices, to which mHealth technologies broadly belongs, mHealth performance mainly depends on whether both patients and clinicians are actively involved in its use [18-21]. However, current evidence has not addressed oncologists, and little is known about what incentivizes their use of mobile technologies; although oncologists have previously been shown to be open, in principle, to considering mHealth technology as part of patient care [22].

### Objective

In summary, the interest in the use of mobile apps in cancer care is increasing, but there is little empirical insight into stakeholders’ perceptions. Therefore, to gain insight into key stakeholders’ perceptions of the value that mHealth app use creates, we distributed 2 surveys targeting 2 populations of mHealth app stakeholders—randomly selected cancer clinicians and patients who use internet-enabled mobile devices, such as smartphones. Through these surveys, we gathered data on the use of mHealth apps by patients and on how clinicians and cancer patients perceive the value of mHealth app use. In this study, we therefore aimed to describe the physician and patient population that utilizes mHealth in cancer care, the activities they perform, as well as the reasons for not using it.

## Methods

### Survey Design and Settings

To investigate the use of mHealth in cancer care by patients and clinicians and the reasons for its use, we conducted a cross-sectional, international survey from July 2015 to February 2016. The survey included the European Big 5, that is, France, Germany, Italy, Spain, and the United Kingdom, as well as the United States. These countries exhibit some of the highest smartphone ownership rates and have mobile broadband penetration rates above 75% [23]. Concurrently, although cancer is now on the rise in developing countries, the overall age-standardized cancer rate is still approximately 1.8 times higher in more developed countries [24]. Thus, the diffusion and the current performance of a health care innovation device such as mHealth can suitably be investigated in these countries, ensuring the ecological validity of the study. The first draft of the survey was based on existing literature and previous surveys and experiments on mHealth [25-27]. The survey questions were finalized by the authors and translated into Spanish, French, German, and Italian by professional medical editors in the different languages. To guarantee the accuracy of the translations, we pilot tested them with a group of clinicians and patient representatives. A final completeness check was implemented, and all essential items were made mandatory; when possible, a nonresponse option such as *not applicable* was provided. In the final version of the survey, we asked clinicians and patients up to 37 and 32 questions, respectively (some on the questions were dependent on previous answers).

### Study Population

Eligible clinicians included oncologists who used smartphones and other devices for internet access, although not necessarily for mHealth. Similarly, eligible patients were those diagnosed with any kind of cancer who owned a smartphone that could access the internet. The survey was administered through qualtrics^XM^, a US-based company established in 2002 that allows researchers to conduct surveys in communities that are traditionally hard to reach. To construct the panel, potential respondents were recruited through the Web (using specific keywords) and outlined based on their characteristics, and a stratified sample was later invited to join the research panel.

In particular, patients were selected based on a population panel that provides recruitment via Web (Web banners, pay per click, natural optimization of research, affiliate marketing, email, and online public relations activities). Oncologists were reached out drawing on panels that are constructed by telephone recruitment or via recruitment portals starting from specialized databases—such as those of scientific communities.

The survey was sent to 1800 oncologists and 1800 cancer patients consisting of a random sample of panelists stratified by country and age group. Both clinicians and patients were invited to participate in the study via email through Qualtrics and were provided with a link leading to the survey. The main screen of the online questionnaire provided all respondents with the aim of the study, the investigator information, and the expected time length of the survey (approximately 10 min). The respondents’ right to confidentiality was respected, and consent to participate in the survey was obtained.

One concern when using online recruitment panels is that subjects rush through the online questionnaire without properly reading the provided instructions and questions. To increase the statistical power and reliability of our dataset, we screened respondents based on several criteria. First, we included control questions to detect spammers. Those study participants who failed to answer the control questions, answered all the questions in the same way, or filled in boxes with no-sense comments were excluded from our sample (28 clinicians and 68 patients). Second, we examined the time subjects took to fill out the questionnaire (for clinicians, a mean of 6.31 min and for patients, 6.40 min). Extreme deviations from the average time to complete the questionnaire were treated as outliers and excluded from further analysis. Thus, respondents within the lowest 1% percentile (less than 2.5 min) in terms of total time till survey completion were excluded. Furthermore, we checked whether the subjects’ internet protocol (IP) addresses overlapped. In such cases, duplicate entries from the same IP address were excluded from our analysis (12 respondents—patients—in total).

### Variables

The survey instrument included 4 different domains in both the clinician and patient versions: (1) sociodemographic variables (age, sex, education, and salary level), (2) mHealth utilization, (3) mHealth activities performed, and (4) reasons for not using mHealth. Both clinicians and patients were asked about their use of mHealth technologies for the management of cancer. Users, namely, individuals who owned a smartphone or any other mobile device and who used it for cancer-related purposes, were then asked to report for what purposes they used mHealth by choosing from a list of activities. These activities related to different degrees of pervasiveness of the technology aimed at highlighting different user expertise levels, based on a previously designed framework by Nasi et al [10]. As a result, respondents were further classified as either basic (ie, those who used mHealth to schedule appointments, access personal health care information, or read test results only) or advanced users (ie, people who used mHealth to monitor treatment side effects and prevent further events). In contrast, respondents not using mHealth were asked to identify the reasons that had so far hindered them from adopting the technology using 5-point Likert scale items in the following format: 1=I completely disagree, 2=I disagree, 3=I neither disagree nor agree, 4=I agree, and 5=I completely agree.

### Statistical Analysis

Descriptive statistics were used to report respondents’ sociodemographic information and the degree of utilization of mHealth in managing cancer care. To measure the relation between specific sociodemographic information and the likelihood of being mHealth users, 2 possible sources of sample selection bias need to be addressed. First, as the survey was administered online, the results are influenced by the general digital divide in the population. Second, the survey was completed only by patients and clinicians using mobile technologies for any purpose, ie, if the respondent could access the internet but was not a user of mobile technologies (eg, smartphones or tablets), the survey was concluded, and no further questions were asked. In the first case, the sample selection bias is relevant but does not influence our results as the target population of mHealth technologies does not include people without basic technological endowments (eg, a computer with internet access). The second source of bias, instead, is more relevant because it refers to the population having access to the Web but whose mobile endowment is low. Ideally, we should not exclude these respondents as they are a part of the potential target of mHealth. In our sample, only 21 out of 2170 respondents reported not using mobile technologies for any given purpose. To account for this potential bias that could still have a potential effect on our results, we used 2 different statistical approaches, namely, a propensity score–based regression adjustment (PSBRA) with weighting and a Heckman probit selection model (HPSM). For both the propensity score equation in the PSBRA and the selection equation in the HPSM, the independent variables were the age group, nationality, and salary level of the respondent. The choice of using 2 different procedures was motivated by the necessity of testing the robustness of the obtained estimations because of the disproportion between censored and uncensored observations. Analyses were conducted with STATA software, version 14 (Stata Corp).

### Workshop and Stakeholder Engagement

The main results from the survey were shared with several stakeholders to solicit input and feedback as well as develop policy recommendations for an appropriate spread of mobile technologies. An international workshop was organized in Milan, Italy, to facilitate interaction with over 100 stakeholders, including patients, clinicians, app developers, the pharmaceutical and medical technology industry, telecom industries, experts in medical communications and health education, payers, and policymakers.

We announced the international workshop through different channels: (1) the general way, that is, by using the website and social networks normally used by our university (Bocconi University) to promote events and (2) a more specific way, that is, by compiling a mailing list of all potential stakeholders at the international and national levels. Participation was free of charge and travel expenses were covered by participants.

The session was intended to focus on the discussion of the results arising from the survey. Specifically, 3 main questions were aimed toward participants: (1) why patients and clinicians do not use mHealth evenly, (2) what are the main barriers that have slowed the adoption of mHealth in cancer care, and finally (3) what is the likely effect of mHealth on clinicians’ activity and on patients’ quality of life. A member of the research team facilitated the workshop, ensuring the surfacing of diverse perspectives and a rich discussion of issues. The feedback from the workshop was used to develop a set of questions that we posed to an expert roundtable.

The roundtable consisted of 4 participants who represented 2 leading patient and clinician associations: European Cancer Patient Coalition and the European School of Oncology in Europe and Healthwise Organization and the Multinational Association of Supportive Care in Cancer in the United States. The discussion was moderated by a member of the research team. Both the workshop and the roundtable were recorded and professionally transcribed.

## Results

### Study Population

Valid responses were obtained from 1116 of the clinicians surveyed (62.00% response rate) and 1033 of the cancer patients interviewed (57.39%). The respondents’ characteristics are summarized in Tables 1 and 2. The patients were mostly female (637/1033, 61.66%) and aged over 45 years (798/1033, 77.25%), whereas the clinicians were mostly male (795/1116, 71.24%) and evenly apportioned between the 2 age groups. With respect to education, 28.46% of patients (294/1033) had received no education or had only attended primary school, 37.37% (386/1033) had either completed secondary school or achieved an undergraduate degree, and the remaining 34.17% (353/1033) had completed graduate (18.0%) or postgraduate (16.2%) education. Approximately one-third (335/1033, 32.43%) of the patients were employed full time, 12.88% (133/1033) were employed part time, and about one-third (366/1033, 35.43%) were retired. Employed patients prevalently earned less than US $30,000 per year (178/1033, 17.23%) or between US $30,001 and US $50,000 per year (129/1033, 12.49%). In contrast, more than half of the clinicians (721/1116, 64.61%) earned over US $75,000 per year, with relevant observed differences between Germany, the United Kingdom, and the United States (62.7%, 70.4%, and 91.1%, respectively) and Mediterranean countries (30.5% in France, 26.0% in Italy, and 7.7% in Spain).

**Table 1:** Patient sample characteristics by country, 2016.

Patient characteristics		France (n=103)	Germany (n=101)	Italy (n=105)	Spain (n=102)	United Kingdom (n=111)	United States (n=511)	Total (N=1033)
**Sex, n (%)**								
	Male	35 (34.0)	45 (44.6)	39 (37.1)	37 (36.3)	53 (47.7)	187 (36.6)	396 (38.33)
	Female	68 (66.0)	56 (55.4)	66 (62.9)	65 (63.7)	58 (52.3)	324 (63.4)	637 (61.67)
**Age group (years), n (%)**								
	Under 45	20 (19.4)	24 (23.8)	31 (29.5)	41 (40.2)	13 (11.7)	106 (20.7)	235 (22.75)
	Over 45	83 (80.6)	77 (76.2)	74 (70.5)	61 (59.8)	98 (88.3)	405 (79.3)	798 (77.25)
**Education level, n (%)**								
	No or primary education	29 (28.2)	57 (56.4)	56 (53.3)	8 (7.8)	28 (25.2)	110 (21.5)	288 (27.88)
	Secondary or undergraduate education	26 (25.2)	24 (23.8)	8 (7.6)	34 (33.3)	32 (28.8)	262 (51.3)	392 (37.95)
	Graduate	30 (29.1)	7 (6.9)	29 (27.6)	48 (47.1)	30 (27.0)	42 (8.2)	186 (18.01)
	Postgraduate	18 (17.5)	13 (12.9)	12 (11.4)	12 (11.8)	15 (13.5)	97 (19.0)	167 (16.17)
**Employment status, n (%)**								
	Full-time employed	32 (31.1)	29 (28.7)	43 (41.0)	57 (55.9)	28 (25.2)	146 (28.6)	335 (32.43)
	Part-time employed	13 (12.6)	19 (18.8)	16 (15.2)	5 (4.9)	15 (13.5)	65 (12.7)	133 (12.88)
	Unemployed	6 (5.8)	5 (5.0)	6 (5.7)	15 (14.7)	1 (0.9)	21 (4.1)	54 (5.23)
	Not employed and not looking for work	4 (3.9)	5 (5.0)	1 (1.0)	7 (6.9)	9 (8.1)	26 (5.1)	52 (5.03)
	Unable to work	6 (5.8)	9 (8.9)	7 (6.7)	4 (3.9)	7 (6.3)	50 (9.8)	83 (8.03)
	Student	0	0	3 (2.9)	1 (1.0)	0	6 (1.2)	10 (0.97)
	Retired	42 (40.8)	34 (33.7)	29 (27.6)	13 (12.7)	51 (45.9)	197 (38.6)	366 (35.43)
**Salary level, n (%)**								
	≤US $30,000	23 (22.3)	20 (19.8)	41 (39.0)	42 (41.2)	19 (17.1)	33 (6.5)	178 (17.23)
	US $30,001-US $50,000	16 (15.5)	14 (13.9)	13 (12.4)	18 (17.6)	14 (12.6)	54 (10.6)	129 (12.49)
	US $50,001-US $75,000	6 (5.8)	10 (9.9)	4 (3.8)	0	6 (5.4)	52 (10.2)	78 (7.55)
	>US $75,001	0	4 (4.0)	1 (1.0)	2 (2.0)	4 (3.6)	72 (14.1)	83 (8.03)
	Missing or not applicable	58 (56.3)	53 (52.5)	46 (43.8)	40 (39.2)	68 (61.3)	300 (58.7)	565 (54.70)

**Table 2:** Clinician sample characteristics by country, 2016.

Sample characteristics		France (n=105)	Germany (n=150)	Italy (n=123)	Spain (n=104)	United Kingdom (n=108)	United States (n=526)	Total (N=1116)
**Sex, n (%)**								
	Male	75 (71.4)	116 (77.3)	81 (65.9)	55 (52.9)	74 (68.5)	394 (74.9)	795 (71.24)
	Female	30 (28.6)	34 (22.7)	42 (34.1)	49 (47.1)	34 (31.5)	132 (25.1)	321 (28.76)
**Age group (years), n (%)**								
	Under 45	62 (59.0)	49 (32.7)	44 (35.8)	66 (63.5)	70 (64.8)	286 (54.4)	577 (51.70)
	Over 45	43 (41.0)	101 (67.3)	79 (64.2)	38 (36.5)	38 (35.2)	240 (45.6)	539 (48.30)
**Education level, n (%)**								
	No or primary education	0	0	0	0	0	0	0
	Secondary or undergraduate education	0	0	0	0	0	0	0
	Graduate	0	0	0	0	0	0	0
	Postgraduate	105 (100.0)	150 (100.0)	123 (100.0)	104 (100.0)	108 (100.0)	526 (100.0)	1116 (100.00)
**Employment status, n (%)**								
	Full-time employed	105 (100.0)	150 (100.0)	123 (100.0)	104 (100.0)	108 (100.0)	526 (100.0)	1116 (100.00)
	Part-time employed	0	0	0	0	0	0	0
	Unemployed	0	0	0	0	0	0	0
	Not employed and not looking for work	0	0	0	0	0	0	0
	Unable to work	0	0	0	0	0	0	0
	Student	0	0	0	0	0	0	0
	Retired	0	0	0	0	0	0	0
**Salary level, n (%)**								
	≤US $30,000	3 (2.9)	4 (2.7)	13 (10.6)	6 (5.8)	1 (0.9)	5 (1.0)	32 (2.87)
	US $30,001-US $50,000	28 (26.7)	14 (9.3)	40 (32.5)	48 (46.2)	10 (9.3)	4 (0.8)	144 (12.90)
	US $50,001-US $75,000	41 (39.0)	34 (22.7)	37 (30.1)	42 (40.4)	20 (18.5)	31 (5.9)	205 (18.37)
	>US $75,001	32 (30.5)	94 (62.7)	32 (26.0)	8 (7.7)	76 (70.4)	479 (91.1)	721 (64.61)
	Missing	1 (1.0)	4 (2.7)	1 (0.8)	0	1 (0.9)	7 (1.3)	14 (1.25)

### Patient and Clinician Usage of Mobile Health

Of the 2149 participants surveyed, 1153 (53.65%) had previously accessed some sort of mobile technology for cancer-related purposes. Different mHealth access rates were observed in the 2 end-user groups. Among patients, 28.46% (294/1033) were mHealth users: nonusers were the majority in all countries assessed, although there were between-country differences. Clinicians, in contrast, were most often mHealth users: 76.97% of the respondents (859/1116) utilized mobile technology in their daily activity or in the management of cancer patients. The highest percentage was observed in the United States (459/526, 87.26%). Regarding the intensity of use, we observed that among clinician respondents, 32.26% (360/1116) were advanced users and 44.71% (499/1116) were basic users, whereas 18.39% (190/1033) of patients reported being advanced users versus 10.16% (105/1033) of basic users (Table 3).

**Table 3:** Distribution of users and nonusers of mobile health in the analyzed countries, 2016 (N=2149).

Users			France	Germany	Italy	Spain	United Kingdom	United States	Total
**Patients, total**			**n=103**	**n=101**	**n=105**	**n=102**	**n=111**	**n=511**	**N=1033**
	**Users, n (%)**		16 (15.5)	34 (33.7)	46 (43.8)	25 (24.5)	18 (16.2)	155 (30.3)	294 (28.46)
		Basic users	8 (7.8)	10 (9.9)	15 (14.3)	9 (8.8)	6 (5.4)	56 (11.0)	104 (10.07)
		Advanced users	8 (7.8)	24 (23.8)	31 (29.5)	16 (15.7)	12 (10.8)	99 (19.4)	190 (18.39)
	Nonusers, n (%)		87 (84.5)	67 (66.3)	59 (56.2)	77 (75.5)	93 (83.8)	356 (69.7)	739 (71.54)
**Clinicians, total**			**n=105**	**n=150**	**n=123**	**n=104**	**n=108**	**n=526**	**N=1116**
	**Users, n (%)**		72 (68.6)	104 (69.3)	72 (58.5)	60 (57.7)	92 (85.2)	459 (87.3)	859 (76.97)
		Basic users	33 (31.4)	38 (25.3)	35 (28.5)	36 (34.6)	56 (51.9)	301 (57.2)	499 (44.71)
		Advanced users	39 (37.1)	66 (44.0)	37 (30.1)	24 (23.3)	36 (33.3)	158 (30.0)	360 (32.26)
	Nonusers, n (%)		33 (31.4)	46 (30.7)	51 (41.5)	44 (42.3)	16 (14.8)	67 (12.7)	257 (23.03)

### Clinicians’ and Patients’ Mobile Health Activities

Among the patients classified as mHealth user, approximately half of the respondents used mobile technologies for automation and decision-making support into activities such as scheduling an appointment (157/294, 53.4%), accessing personal information (147/294, 50.0%), and reading test results (135/294, 45.9%). Only approximately one-third of users and, therefore, about one-tenth of total patient respondents, supported treatment and follow-up phases through mHealth by either monitoring side effects (108/294, 36.7% of users), helping prevent further events (85/294, 28.9%), or taking medications as prescribed (97/294, 33.0%; Table 4). With regard to clinicians, the majority accessed mHealth to carry out activities that pertain to the automation and interaction domains: 88.6% (761/859) used mobile apps to perform literature research, 66.9% (575/859) to interact with their colleagues, and 44.6% (383/859) to communicate directly with patients. Fewer clinician users utilized mHealth for decision-making purposes: 46.1% (396/859) used mobile apps to access patients’ electronic health records, 44.0% (378/859) to collect test results, and a smaller number (324/859, 37.7%) of users used mHealth to support decision making for ordering further tests. A minority of users performed activities that support treatment and follow-up in the care process, such as side-effect management (318/859, 37.0%) and compliance monitoring (116/859, 13.5%; Table 5).

**Table 4:** Activities performed by patient users, by degree of pervasiveness of the technology.

Activities performed by patient users		Frequency of the activity among users (n=294), n (%)	Frequency of the activity among total respondents (N=1033), n (%)
**Activities supporting automation and interaction**			
	Schedule an appointment with a physician	157 (53.4)	157 (15.20)
**Activities supporting decision making processes**			
	Access personal health care information	147 (50.0)	147 (14.23)
	Get test results	135 (45.9)	135 (13.07)
**Activities supporting treatment and follow-up**			
	Monitor side effects (nausea, vomiting, and diarrhea)	108 (36.7)	108 (10.45)
	Help prevent further events (cancer progression and recurrence)	85 (28.9)	85 (8.23)
	Help in taking medications as prescribed	97 (33.0)	97 (9.39)

**Table 5:** Activities performed by clinician users, by degree of pervasiveness of technology.

Activities performed by clinician users		Frequency of the activity among users (n=859), n (%)	Frequency of the activity among total respondents (N=1116), n (%)
**Activities supporting automation and interaction**			
	Literature research	761 (88.6)	761 (68.19)
	Communicate directly with patients	383 (44.6)	383 (34.32)
	Interact with colleagues for timely decision-making	575 (66.9)	575 (51.52)
**Activities supporting decision-making processes**			
	Access patients’ electronic health records	396 (46.1)	396 (35.48)
	Get test results	378 (44.0)	378 (33.87)
	Decision support for ordering further tests	324 (37.7)	324 (29.03)
**Activities supporting treatment and follow-up**			
	To monitor compliance (principal treatment)	116 (13.5)	116 (10.39)
	To manage side effects	318 (37.0)	318 (28.49)

### Professional Mobile Health Divide Between Clinicians and Patients

Table 6 shows the marginal probabilities of use at means that were 69.8% for clinicians and 38.7% for patients using the PSBRA technique (69.5% and 38.7%, respectively, using HPSM). Other things being equal, clinicians use mHealth more than patients, thus, highlighting an inefficient activation of the complementarities between the two main actors involved in the process of care. Age and salary level influenced mHealth adoption in both end-user groups. Among clinicians, younger professionals exhibited an approximately 15 percentage point higher likelihood of being mHealth users (82.9% vs 64.6% using PSBRA), while this gap was even wider for patients, verging on 30 percentage points. Salary level had a similar impact, with more affluent respondents more likely to be mHealth users than the less well-off respondents. These variables also explain the width of the divide between clinicians and patients. With respect to age, the divide was significantly higher for old respondents (35.4% using PSBRA) than that for young respondents (24.5%), whereas in regard to salary level, the divide was lower for low-income (29.5% using PSBRA) and high-income categories (23.2%) and significantly higher for medium-income ones (between 36.4% and 43.7%). Further differences arose when country-level situations were addressed, with the divide being as high as nearly 50 percentage points in the United Kingdom (51.7% using PSBRA).

**Table 6:** Marginal probabilities of mobile health use (N=2149).

Statistical approaches^a,b^		PSBRA^c^, %			HPSM^d^, %		
		Clinicians	Patients	Divide	Clinicians	Patients	Divide
Main effect		71.8	40.2	31.6	71.2	39.6	31.6
**Age (years)**							
	≤45	82.9	58.5	24.5	80	54.2	25.7
	>45	64.6	29.2	35.4	66	30.6	35.4
**Salary**							
	≤US $30,000	63.7	34.2	29.5	61.2	33.3	28
	US $30,001-US $50,000	70.6	34.3	36.4	70.1	34.4	35.7
	US $50,001-US $75,000	75.5	31.9	43.7	75.2	30.2	44.9
	>US $75,000	74	50.8	23.2	73.7	50.5	23.1
**Country**							
	France	62	27.5	34.5	60.6	25.5	35.1
	Germany	60	43.4	16.6	64.4	47	17.4
	Italy	50.6	51.4	−0.8^e^	55.2	55.5	−0.3^e^
	Spain	46	26.3	19.7	49.5	29.3	20.2
	United Kingdom	75.5	23.8	51.7	77	28	48.9
	United States	84.7	46.2	38.5	82.1	41.6	40.5

^a^Marginal probabilities at both values of *clinician/patient* dummy are displayed.

^b^The regression used to estimate propensity scores had a pseudo-R-squared value of 0.15 and the goodness-of-fit test showed a Pearson chi-square value of 19.1. The logit model included the propensity score as covariate and as probability weight.

^c^PSBRA: propensity score–based regression adjustment with weighting.

^d^HPSM: Heckman probit selection model.

^e^Not significant.

### Reasons That Hinder Greater Mobile Health Use

Participants who did not belong to the user category were asked about their concerns regarding mHealth use and answered 5-point Likert scale items (Table 7). On the patient side, the most diffused concerns pertained to the preference for traditional means of communication with their doctor (mean 4.26, SD 0.93), the lack of knowledge about the potentials of information technologies (mean 3.82, SD 1.17), and the doubts about the reliability and effectiveness of mHealth for medical purposes (mean 3.03, SD 1.07). For nonuser clinicians, the most substantial doubts were related to the preference for in-person visits (mean 4.13, SD 0.91) and the inability of patients to use smartphones (mean 3.44, SD 0.94).

**Table 7:** Barriers for mobile health (mHealth) use rated on a 5-point Likert-type scale by patient and clinician nonusers.

Participant, barrier		Mean (SD)
**Patient nonusers**		
	I am worried about the protection of the confidentiality of my personal, medical and health information	2.65 (1.21)
	I do not trust the technical reliability of the software	2.86 (1.06)
	I think mobile technologies are not effective and reliable for medical purposes	3.03 (1.07)
	I am not attracted by mHealth because I cannot use the devices properly	2.65 (1.08)
	I prefer to communicate and meet my doctor in person	4.26 (0.93)
	I was not aware of this possibility	3.82 (1.17)
	I cannot afford the costs of mobile devices and connection	2.64 (1.23)
**Clinician nonusers**		
	I am doubtful about providing mobile type of support because of data security concerns	2.89 (1.18)
	I do not trust the technical reliability of the software	2.34 (0.95)
	I am not interested in mHealth because I cannot use the devices properly	2.12 (0.97)
	I was not aware of this potential use of mobile phones	3.02 (1.17)
	I think mobile technologies are not effective and reliable for medical purposes	2.22 (0.96)
	I realized patients are often not able to utilize mobile technologies	3.44 (0.94)
	I still prefer to communicate and meet my patient in person	4.13 (0.91)
	I think it would be uncomfortable mixing the face-to-face relationship with my patients with the virtual practice produced by mHealth	2.90 (1.13)

### Qualitative Feedback

The large spectrum of stakeholders involved in the workshop helped identify further key themes. These were the generic nature of medical apps, the lack of user-friendliness because of integration into work and life contexts, the poor interaction interfaces, and the confusion about whether and when medical apps must be considered medical devices and whether they must meet evidential requirements or not. During the roundtable, the experts agreed that current apps are seldom developed with patients in mind and that, in many cases, the app functionalities do not meet patients’ expectations and needs. Therefore, participants agreed that it would be extremely important to identify the target audience’s wishes or expectations before designing and developing new apps. In particular, the participants emphasized that to define the content of apps, it would be fundamental to understand the characteristics of the main target population (ie, old/young user, type and stage of disease, and different familiarity levels with technology), the language (the simpler the better, avoiding scientific language, and making the app immediately easy to use), and the layout (ie, small fonts on a small screen are a barrier for old people).

## Discussion

### Principal Findings

With the aging of the population and the epidemics of chronic diseases, the financial sustainability of health care systems across the globe is at threat and calls for new paradigms where patients are empowered to stay healthy and/or to self-manage their conditions and hospitals only serve to treat the acute phases of diseases and to connect the community and patients’ home to deliver long-term chronic care. In such a context, mHealth, leveraging on the increase in mobile smartphone subscribers (over 4.4 billion in 2017 [28], representing over 2 out of 3 adults on earth), is emerging as a viable solution to keep patients informed and empowered, to provide clinicians with timely data that can improve their capacity to assess patients’ health status, and to help improve hospitals to reorganize their production function and management processes to better fit the evolving needs of the population [29].

Cancer survivors experience differing needs in terms of medical care, psychosocial support, and practical needs of daily living, and mHealth apps can provide access to information and health behavior interventions that are low cost, easy to access, and personalized to their specific needs [30]. Benefits can go as far as increasing a patient’s chances of overall survival [31]. However, despite the largely acknowledged potential and the increase in artifact development, available evidence on the actual use of mobile technologies in cancer care and cancer supportive care is still scant. This study found a utilization rate of mHealth of less than 30% by cancer patients. These results are slightly higher, although in the same order of magnitude, compared with those of a cross-sectional survey administered at a University Hospital in Spain, according to which 20.3% of the surveyed hematology-oncology patients had a health app [13]. Clinicians, in contrast, exhibited a more widespread utilization of mHealth according to our survey results, with over three-quarters of users among those surveyed.

In any case, not all users accessed mHealth for the same purposes; our analysis identified a further gap, the one between basic and advanced users.

Clinicians tend to use mHealth mainly in isolation, without mHealth-based interaction with patients. Indeed, clinician users reported that they access mHealth extensively to perform activities that support automation and data collection (such as *Interaction with colleagues* and *Literature research*) and less often to support clinical decision making.

Our survey results are thus consistent with previously published studies that highlighted a limited focus of mHealth experimental studies and apps on treatment and follow-up activities in the oncology field [9,10].

Several barriers still halt wider adoption by both clinicians and patients, the main one being the preference for in-person communication and the related concern that mobile technologies might hinder the relationship of trust. Both nonuser groups support these apprehensions, and our results are akin to previous literature results that identified the wish for personal contact with the treating physician as the main reason for app refusal in a cancer patient survey [14] as well as the clinician fear that mHealth might jeopardize the patient-clinician relationship and increase their workload [29]. However, according to a broader systematic, narrative review, after adopting mHealth apps, patients felt empowered and perceived a positive impact on the relationship with their providers [32].

On a different note, our analysis confirmed that, as is seen for the use of internet and smartphones in general, age, education, and income play important roles in explaining the use of mHealth in cancer care by both clinicians and patients. However, other things being equal, we found that the use of mHealth technologies is significantly more common among clinicians than among patients and that factors such as age, income, and origin further contribute to modulating the extent of this divide. This divide might be present because mHealth, such as most types of health technologies (eg, medical devices), represents a work instrument for clinicians who, for the sake of improving their performance, normally are prone to and represent the natural target for technological innovation [33,34]. However, consumers do not normally encounter health technologies until they become patients and, in principle, would not care at all about them unless they happened to contract a disease or were prescribed the technology by their doctors. Much is known about the typical agency relationship that happens between patients and doctors together with the supplier-induced demand that makes patients’ consumption of health care services highly dependent upon doctors’ advice [13,35,36]. Moreover, there is evidence that the membership in interprofessional alliances and networks for change is instrumental to facilitate or hinder the diffusion process of new technologies [37]. Physicians participate in specific networks for change that place them in a privileged position in the diffusion of innovations. Until the sociodemographic evolution alleviates this trend, clinicians might play decisive roles in spreading mHealth utilization in cancer care and recruiting more and more patients to adopt it.

In fact, this professional divide represents a barrier to mHealth effectiveness in cancer treatment. If the promise of mHealth is to be fulfilled, clinician and patient usage rates will need to converge. There is merit in incentivizing oncologists to adopt cancer apps in routine practice to encourage patients to access mHealth at greater length.

### Incentives for Greater Mobile Health Utilization

To enhance clinician use, several different layers can be approached. The first dimension pertains to artifact design; ideally, cancer apps would need to be designed in such a way that would strengthen the patient-clinician relationship, and they should be tested for validity, accuracy, and self-efficacy to help clinicians and patients orient themselves in what now seems to be an app overload [38].

App designers and developers must do more to bring their end users into the design process. Our findings point to the need for app developers to leverage toolkits to enable patients and physicians to more fully engage in the design and development process, each contributing with their own expertise [39]. This will enable the cocreation of solutions. In working toward the development of a sustainable Information Technology system, it is important to engage the final users, particularly the clinicians and patients, throughout the different phases from problem identification to the design and development phase, aligning the project trajectory to users’ needs and expectations and providing clinicians with the opportunity for self-reflection and revisions. Unfortunately, many mHealth apps are designed without considering the needs of their users in terms of either patients or clinicians [4]. The literature lacks empirically validated guidelines or process models on how to design apps with stakeholders rather than for stakeholders. As a result, a recent overview of systematic studies by Byambasuren et al revealed that most mHealth apps are of low quality [40], which hinders their recommendations by clinicians and their use by patients. In cancer care only, Brouard et al evaluated 117 apps for oncological information and treatment monitoring [41] and found that the validation of those apps was generally poor (27.4%).

First of all, these results suggest that designers and developers need to recognize that a one-size-fits-all approach will not work when it comes to apps dealing with conditions such as cancer. Specific apps that account for differences in types of patients, variance in the stage of the disease, and the kind of care one is receiving, as well as the expertise one has with using mobile technologies, will have better chances for increasing adoption and regular usage.

Second, most health apps lack evidence of clinical effectiveness and do not undergo a formal validation and evaluation process [42]; the lack of evidence on whether and under what conditions mHealth delivers on its promise to improve patients’ health outcomes and the efficiency of the health care process further contributes to restraining greater utilization [29,43-45]. More cancer apps need to be tested for their efficacy as, with few exceptions [31,46,47], the evidence base in support of mHealth technologies is still lacking [9]. However, given that the overall performance of mHealth apps is multidimensional, that is, it can be measured from different perspectives (eg, patients, caregivers, and clinicians), it is necessary to develop a methodological framework to include a wider array of benefits beside clinical outcomes. This is part of the objectives of Pushing the boundaries of Cost and Outcome Analysis of Medical Technologies, a large, 3-year, European Union funded project whose recommendations on how to assess mHealth apps are expected in 2020 [48].

Also, the National Institute for Health and Care Excellence in the United Kingdom has recently started a project aimed at providing guidance on the assessment of mobile technologies (ie, *Behaviour change: digital and mobile health interventions*) expected to be delivered in 2020 [49].

Third, efficient regulation can help promote the adoption of mHealth apps. mHealth apps are classified as medical devices when they are used for diagnosis, prevention, treatment, monitoring, or alleviation of disease in human beings and for this reason must respond to high regulatory standards for demonstrating clinical benefit and safety [50]. Nevertheless, regulatory systems have rarely been able to catch up with the exponential launch of mHealth apps in the global market and have often been equivocal in establishing whether a software-based technology has to be treated as a medical device. This resulted in a very poor number of clinical trials that included digital health technologies, 860 worldwide in 2017 [51] compared with the number of mHealth apps for managing health conditions in the same period (126,000) [3], which means that the large majority of mHealth apps have entered the market without any clinical evidence in support [52]. This might have reduced clinicians’ and ultimately patients’ confidence in the reliability and efficacy of the apps. The US Food and Drug Administration (FDA) [53] and the Directorate for Health and Consumers of the European Commission [54] have long been trying to clarify the regulatory standards that digital health technologies need to meet and what evidential requirements need to be developed by app manufacturers. Furthermore, the FDA has led a working group within the International Medical Device Regulatory Forum aimed at harmonizing the regulatory framework for software-based technologies across different jurisdictions [55] and will include in the fiscal year 2019 budget a Center of Excellence for Digital Health that will aim at modernizing the regulatory approach to digital health [56]. These efforts are crucial as they would guarantee common rules to manufacturers that work in a global environment and would increase the level of trust in end users.

Fourth, data protection must be addressed to increase end users’ confidence in using mHealth apps. Clinicians still do not feel the full reassurance about the reliability of the data collected and of the available apps [57]. Data should not only be reliable but their usability is also particularly critical: the vast amounts of data potentially available to patients and providers could easily overwhelm them if not put to best use. The FDA has published premarket and postmarket guidance that offers recommendations for the comprehensive management of medical device cybersecurity risks and continuous improvement throughout the total product life cycle as well as incentivized changing marketing and distributed medical devices to reduce risks [58]. More recently, the General Data Protection Regulation, a European Union law, aimed at regulating personal data in the digital world [59]. Although they are too new to be assessed, we think these efforts go in the right direction of increasing clinicians’ and consequently patients’ confidence in using mHealth apps.

Finally, innovative, multidisciplinary home-based models of care are now available for cancer patients who can be actively maintained with oral anticancer drugs and have shown preliminary success in optimally managing adherence during pilot testings [60-62]. Although the impact of personalized mHealth apps on adherence and other significant outcomes of patients on oral anticancer medications is yet to be assessed [63], appropriate economic incentives and related formulas are needed to spur their utilization of these devices [20].

In conclusion, like all other medical devices, mHealth uptake and diffusion largely depend on clinicians’ conviction, but, differently from some other medical devices (eg, implantable devices), the effectiveness of mHealth heavily depends upon patients playing an active role and using it at the same pace as clinicians.

### Study Strengths and Limitations

This is the first survey including a large, international sample size comparing 6 different countries in North America and Europe and, even more importantly, covering the two most important end users of mHealth: patients and clinicians. In fact, albeit scant, previous research has primarily addressed cancer patient needs and attitudes toward mobile technologies and not those of clinicians. Although past estimates exist for other specializations [64], to our knowledge, this represents the first evidence of mHealth utilization by clinicians in the cancer field. Second, this is the first study that combines the survey approach with a more qualitative method (workshop and roundtable with key stakeholders) to better interpret and complement the quantitative evidence emerging from the survey to ultimately provide concrete recommendations to decision makers.

However, this study suffers from some limitations that should be considered in subsequent studies. First, the study was based on a volunteer online access panel and, thus, is not entirely representative of the reference population as only individuals who possess some degree of digital competence could be reached and included. However, we believe that the online tool contributed to highlighting the smallest divide between clinicians and patients, which would likely be larger had we not used a digital tool. Moreover, the investigation in the user groups of the activities performed by patients and clinicians who use mHealth was self-reported and not based on actual records of their practice. Finally, this survey presents the limit of generalizability; thus, the divide and the models tested are valid in cancer and cancer supportive care only, and as much as the results are extremely significant, they might not hold true for other types of diseases.

### Conclusions

The use of mobile apps in health and in cancer care is literally booming but poor knowledge exists on who is using mHealth, for what purposes, what kind of apps are used, and what is the likely future of mHealth in clinical practice. In this study, we contributed to filling these gaps: our findings highlight 2 types of digital divides in cancer care—one mediated by socioeconomic and educational inequalities among patients and the other by the rift between how doctors and patients are deploying these technologies. For mHealth to yield its full benefits, it will have to integrate these two ends rather than foment the existing divide.

## Abbreviations

FDA: US Food and Drug Administration
HPSM: Heckman probit selection model
IP: internet protocol
mHealth: mobile health
PSBRA: propensity score–based regression adjustment
